# The Impact of Self-Narratives of Motherhood for Mothers of Children with Autism

**DOI:** 10.3389/fpsyg.2016.01899

**Published:** 2016-12-05

**Authors:** Jerzy Trzebiński, Agnieszka Wołowicz-Ruszkowska, Adrian Dominik Wójcik

**Affiliations:** ^1^Department of Psychology, SWPS University of Social Sciences and HumanitiesWarsaw, Poland; ^2^Department of Education, Maria Grzegorzewska UniversityWarsaw, Poland; ^3^Faculty of Humanities, Nicolaus Copernicus UniversityToruń, Poland

**Keywords:** self-narrative, impact of self-narrative, stories of own motherhood, coping with child’s autism, autism spectrum disorders (ASD), self-narrative of life challenge, self-narrative and coping

## Abstract

The main goal of this study was to identify the impact of a narrative construction of a life challenge - discovering to have a child with autism - on the meaning of life and on resources for coping depending on the challenge’s novelty, i.e., the number of years from the diagnosis. Three hundred and sixty four mothers of children with autism participated in a long-term 3 × 2 experiment. Half of the mothers had children with autism at the age of 9–12 years. For the remaining half, having children with autism was a new and stressful life situation. Their children were 2–3 years old and just diagnosed by a medical center as having autism spectrum disorder. The mothers were assigned to one of three study conditions: they were either asked to write stories of their motherhood or to describe their children’s behavior on a questionnaire or they did not participate in any tasks. One month and then 4 months after this task the participants completed measures of meaning of life and several well-being scales. The results indicated that following the narrative writing the participants had the highest scores on the meaning of life and well-being scales. This affect was sustained over 4 months and was significant only for mothers with older children. The mediation analysis showed that the effects of the experimental conditions on different well-being scales were mediated by the changes in perceived meaning of life. The results suggest that construction of self-narratives of difficult ongoing challenges facilitates meaning making and subsequently strengthens resources for coping. However, it seems that a meaning-making construction of such self-story may be blocked by the uncertainty and stress caused by novelty of the challenging situation.

## Introduction

Many studies revealed that writing about one’s traumatic or negative past experiences provides a variety of psychological and health benefits, including improvement in psychological well-being, social relationships, professional and academic achievements, physiological functioning measured in a variety of ways (Pennebaker, 1993; Pennebaker and Seagal, 1999; Frattaroli, 2006; Burton and King, 2008; Niederhoffer and Pennebaker, 2009), and symptom reduction in patients with chronic illness (Smyth et al., 1999). Research also indicates that the main factor in these positive outcomes is meaning making. Writing or telling about a critical event allows a person to reconstruct or to find some sense in that event, for example as being meaningful to their life. It also seems that a narrative construction of an event, i.e., a specific understanding of the event and its broader context in the frame of a personal story, may be a crucial condition of these outcomes (Bruner, 1990; Baumeister and Newman, 1994; Smyth et al., 2001; Ljubomirski et al., 2006).

The aim of the study was to identify the consequences of a narrative construction of an ongoing challenge, occurring in one’s past, present, and in anticipated future. At the time of the study the participants were in the center of their daily and long-term problems and tasks caused by the challenge. They made decisions, created plans, and emotionally reacted to the ongoing and foreseen events. The participants were mothers of children with autism—and raising a child with this disorder is an example of a difficult life situation. The research questions were: (a) Does a narrative construction of a difficult life situation improve person’s cognitive, motivational, and emotional resources for coping with life’s challenges? (b) What psychological processes evoked by the narrative construction are the base for these changes? Our hypothesis was that there are several interrelated processes which are triggered by the framing of an important life challenge within personal story (Trzebinski, 1998). The self-story context allows a person to build and reinforce meaningful connections between an ongoing challenge and their life (Bruner, 1990; Smith, 1994; Singer, 2004; Pals, 2006). The self-story may thus provide clarity and coherence in understanding one’s goals and the sense of one’s role in the child’s life. It allows a person to take more cognitive control over the past and over the ongoing and foreseen events. This narrative meaning makes a person more trustful in their own abilities and competences, and therefore more hopeful, which results in higher self-esteem, and in hope for success. These processes should facilitate positive emotions in interactions of mothers of children with autism (Brown and Brown, 2003; Schalock, 2004). Better cognitive control and positive emotions facilitate more open and creative thinking about one’s family’s future as well as growing optimism regarding a positive trajectory of one’s child’s development and life in the family. All these factors play a role of mental resources when coping with a challenge.

However, there are some conditions under which the effective narrative meaning making and its consequences can take place. Narrative building of meaningful connections between an ongoing challenge and own life is possible if a person is able to efficiently think and reflect on this challenge. These processes may be disorganized if the challenging situation is too new and too stressful. In such situations, negative emotions, like anxiety and helplessness, may dominate and disorganize higher-order cognitions and cognitive openness (Clarke and MacLeod, 2013; Eysenck, 2013). Unexpectedness and novelty of a challenge should thus moderate the impact of the self-story writing. To test this expectation, we compared the impact of narrative writing in two categories of mothers. One category included mothers who recently received their child’s diagnosis, and therefore were in a situation of stress associated with the unexpected situation. The second category included mothers with a long experience in dealing with the challenge of having a child with autism. The hypothesis was that positive effects of narrative writing would be observed especially or only among mothers of older children.

### The Experience of Motherhood for Mothers with Children with Autism

Having a child with autism spectrum disorder is a difficult challenge in parents’ life. The diagnosis is often followed by intense psychological experiences which may alter woman’s outlook on life and on her personal identity (Emerson, 2003). Raising a child with a disorder can contribute to parenting and family relationships problems (Barnett et al., 2003; Wieland et al., 2014) because it potentially can be a source of two crises (Farber, 1968). The first one is an identity crisis which involves “finding one’s feet” in the role of a parent of a child with autism and of reconciling this role with other social roles. This may be accompanied by a sense of loneliness and alienation, not only in the context of a relationship with others, but also as self-alienation (Graungaard and Skov, 2007; Povee et al., 2012). The second crisis involves the woman’s need to change her idealized image of her child (which she created before the child’s birth) and the expectations that resulted from this image. This is all connected with changing the child’s image from a “healthy child” to that of a child seen and accepted as a “child with a disorder” (Harwood et al., 2007).

The term “autism spectrum disorders” (ASD) is used to describe a group of neurodevelopmental disorders characterized by persistent deficits in social communication and social interaction across multiple contexts, and restricted, repetitive patterns of behavior, interests, or activities (American Psychiatric Association [APA], 2013). These behaviors are a challenge to one’s parenting skills. Compared with parents of children with other disabilities or to parents of typically developing children, parents of children with ASD exhibit higher level of stress (Baker-Ericzén et al., 2005; Hayes and Watson, 2013; van Steijn et al., 2014), anxiety (Hastings et al., 2005), and depression (Singer, 2006; Feldman et al., 2007). High levels of parents’ stress are associated with their child’s social and communicative deficits, problem behaviors (Goin-Kochel and Myers, 2005; Herring et al., 2006; Bishop et al., 2007; Estes et al., 2009), level of dependency (Davis and Carter, 2008), and a wide range of problematic and socially atypical characteristics and behaviors (Hastings, 2003). Additionally, situation of families of children with autism is difficult due to problems with ambivalent and confused public response to their children’s behaviors that lead the parents to avoid participation in social situations together with the child (Marcus et al., 2005). Another negative factor – important especially for families with a very young child – is a lack of an adequate care system for children with ASD and their families, at least in Poland.

A positive adjustment to these changing and growing challenges requires a successive rebuilding and adaptation of the mother’s meaning of life and her vision of the child and their place within the family (Larson, 1998; Lalvani, 2011). These adaptive changes may increase the mother’s well-being and may facilitate her ability to respond sensitively to her child. Improving the mother’s meaning of life and well-being contributes to the mental and physical functioning of a child with ASD (Werner et al., 2009; Baker et al., 2011). The primary expectation of the present research is that the narrative construction of the experience of motherhood for mothers of children with autism spectrum disorder facilitates the meaning making processes and—as a result—strengthens the well-being and other personal resources that are needed when coping with life challenges.

## Materials and Methods

### Participants

A total of 364 mothers of children with ASD who agreed to participate in a three-stage research study were recruited at the Clinical Center for Children with Multiple Disabilities. The study was conducted after all participants understood the study design and signed the informed consent form. The study has been reviewed and approved by the appropriate ethical board. The selection criterion when recruiting the participants was the child’s age and their clinical diagnosis. In the first stage of the study, the mothers were randomly assigned to one of three experimental conditions: narrative, placebo and no-task condition. The children differed in age. Half of them were between 2 and 3 years of age (*M* = 2.19, *SD* = 0.77), and half were between 9 and 12 years of age (*M* = 10.25, *SD* = 1.08). All children were diagnosed as having ASD by the Clinical Center. The participating women had only one child with the ASD diagnosis. The number of children, sex of the diagnosed child, and the level of disability were controlled in data analyses and no significant effects were found.

### Procedure

The participants were first contacted by phone. The experimenter explained the purpose, significance and general time schedule of the research and how the contact information had been obtained. The women were informed that the study would be anonymous, that the results would be used for scientific purposes only and that the main goal of the study was to better understand the situation of families with children with ASD and use this knowledge in the future to help children and their parents. Each woman was also informed that she had the right to withdraw from the study at any time, without any explanation, and that she could ask for additional information and help from the Clinical Center and the research team during the study. For the women who agreed to participate (only 7 women declined), we arranged a personal meeting during which the purpose and methods of the study were explained in more detail. Each woman was presented with a specific research plan according to the experimental condition she had been randomly assigned to. She was asked to create a pseudonym and to use it throughout all stages of the study. Each woman was provided with general instructions on what to do in the first stage and was given an envelope with detailed instructions. She was asked to open the envelope at home and to follow the detailed instructions. The experimenter explained that the questionnaire materials for the second and third stage would be sent by mail. The participants were asked to send the completed questionnaires back within three days in a prepaid envelope that was provided. Their regular visits at the Clinical Center and their meetings with the research assistant helped in maintaining their full participation throughout all the stages of the study.

The first stage of the study only included the participants in the narrative and placebo conditions. In the narrative condition the participants were asked to write a story, in diary form, concerning their child’s situation. The participants were told that writing each day, during a 7-day time period, would be helpful in elaborating the story. The instructions were as follows: “I would like to ask you to write a story about you and your child. A story about your child’s place in your life. Please try to recall events and personal experiences concerning you and your child and then give them a certain wholeness, meaning, by presenting a personal story. You can express your feelings, anxieties, joys, dreams and plans for the future, even those you have had no chance to share with anyone yet.”.

The mothers were instructed to introduce story characters, to write how the story began and what it was about (initial plot exposition). In the following days the participants were asked to write a story in a more elaborated way or as “further chapters”, written in any form and of any length, so that they would feel that the text adequately expressed what had happened and what might happen during their experience of motherhood when having a child with ASD. The length of the obtained texts varied from 2 to 8 pages, all texts had a narrative structure, with the child as a crucial character.

In the placebo condition the participants were asked to answer a long questionnaire including multiple-choice and open questions which were related to the child’s characteristic behavior in specified areas, such as motor skills, communication, self-reliance, socialization, and cognitive abilities. The instructions were as follows: “We are interested in your child’s development in specific areas”. This is why the following questions concern the skills that are related to the given development area. We are intent on obtaining authentic, honest answers. There are no good or bad answers. The examples of questions include: “Please describe situations in which the child alone tends to contact relatives (parents, siblings, grandparents)”, “How does your child use objects: according to or contrary to their purpose?” Each mother had 7 days to recall from her memory or to observe their child’s behavior, and to work on the questionnaires every day. The main goal of the placebo condition was to assure the mothers’ focus, within a 7-day period, on the child’s activity and interactions with family members, without any attempts to create a narrative. The placebo instruction was tested and elaborated during a series of pilot studies. The final results indicated that the mothers responded in line with our expectations: they were focused on recalling and listing specific examples of the child’s behavior, without narrative structuring. In the no-task condition the mothers participated in the second and the third stage only.

In the second and the third stage all participants were asked to fill out a set of questionnaires. The questionnaires were sent by mail to the participants’ home addresses in two packages. In the no-task condition they were divided by a 3-month interval. In the narrative and placebo conditions, after the period of writing had ended, the participants were given packages after 1 month and then after 4 months. The order of the questionnaires in a package was randomly arranged across participants and the time of measurement. All participants were asked to return the questionnaires no later than three days after having received them by using a prepaid envelope addressed to the research team without any sender information on it. The detailed experimental design is presented in supplementary materials (Please see Supplementary Figure S1 for the details).

Two trained and independently working judges have rated the texts written in the narrative condition. They evaluated the level of text content’s organization within a frame of the narrative plot on a 7 points scale (1 – very low; 7 very high). The narrative plot was operationalized as a coherent interconnection of a story protagonist’s intention and complication (Brewer and Lichtenstein, 1981; Lehnert, 1981; Graesser et al., 1994). In case of differences, the judges were instructed to discuss the text and try to reach a consensus, if possible. The final assessments of text narrative coherence was highly consistent – the Two-Way Random Inter Class Correlation for the two judges was.98. The mean of two ratings was used for future analyses.

Although the study lacked the measurement of dependent variables at time 0 it may be argued that fulfilling the well-being scales referring to motherhood experience before the experimental tasks could interfere with these tasks’ influences. Moreover, 2-weeks interval between the measurements may result in the impact of the first measurement on the second one. The mothers were randomly selected to three major experimental conditions and therefore it is highly unlikely that the groups differed significantly at time 0 before taking the Clinical Centre program. Finally, the control group may be treated as a base line for comparisons of efficacy of experimental influence.

A set of six scales measuring the level of meaning in life and resources for coping with life was applied twice in the second and third part of the study. All scales were adapted into Polish and validated in several studies on Polish population.

The set included the following scales:

(1) Purpose in Life Scale (Crumbaugh and Maholick, 1964; Polish adaptation: Życińska and Januszek, 2011) measures the experience of meaning and purpose in life; 20 items, alpha = 0.79, 7-point scale e.g., “My life is…”: 1 – “…out of my hands and controlled by external factors”; 7 – “…in my hands and I’m in control of it”, “When thinking of my life, I…”: 1– “…often wonder why I exist”; 7 – “…always see reasons for being here”. The natural range of scale could vary between 20 and 140 with more positive scores referring to higher levels of purpose in life.
(2) Self-Esteem Scale (Rosenberg, 1965; Polish adaptation: Dzwonkowska et al., 2007), 10 items, alpha = 0.89, e.g., “On the whole, I am satisfied with myself”; (1 – *not at all*, 5 – *always*). The natural range of scale could vary between 10 and 50 with more positive scores referring to higher levels of self-esteem.
(3) Hope Scale (Lopez et al., 2000; Snyder et al., 2000; Polish adaptation: Łaguna et al., 2005) measures the level of hope for success; 12 items, alpha = 0.93, e.g., “I can think of many ways to get out of a jam”; (1 – *definitely false*; 8 – *definitely true*). It includes two subscales: PATHWAYS: belief in having competency to find solutions and AGENCY: belief in having willpower to carry out a plan. The natural range of scale could vary between 12 and 96 with more positive scores referring to higher levels of hope.
(4) Positive and Negative Affect Schedule (Watson and Clark, 1999; Polish adaptation: Fajkowska and Marszał-Wiśniewska, 2009); 10 items, alpha = 0.92. The level of currently experienced positive emotions was measured by PANAS positive affect subscale. The scale consists the names of 10 positive emotions like “cheerful” and participant is asked to evaluate the level of experiencing a given emotion last month (1 – *very slightly or not at all*; 5 – *extremely*). The natural range of scale could vary between 10 and 50 with more positive scores referring to higher levels of positive emotions.
(5) Life Orientation Test–Revised (LOT–R) (Scheier et al., 1994; Polish adaptation: Juczyński, 2009), measures the level of general optimism; 10 items, alpha = 0.88, e.g., “In uncertain times, I usually expect the best”; “I rarely count on good things happening to me (reversed)” (0 – *strongly disagree*; 4 – *strongly agree*). The natural range of scale could vary between 0 and 40 with more positive scores referring to higher levels of general optimism.
(6) Stress Related Growth Scale (Park et al., 1996, modified by Armeli et al., 2001; Polish adaptation: Zieba et al., 2010) adapted to this study, measures the feeling of positive changes in personality as a result of being a mother to a disabled child; 19 items, alpha = 0.83, e.g., “I became more accepting of others” (1 – *decreased strongly*; 5 – *increased strongly*). The natural range of scale could vary between 19 and 95 with more positive scores referring to higher levels of positive changes.

The instructions were adapted to this study by accentuating the present time as a context for estimations of feelings and thoughts.

## Results

We conducted a two-factor ANOVA with repeated measures—3 × 2 × 2: Experimental Condition × Child’s age (younger vs. older) × Time of measurement, for each of the dependent variables. The results share similar patterns. The findings are all summed up in **Table 1**. More detailed descriptive characteristics are presented in **Tables 2**–**7**.

**Table 1:** Summary of ANOVA results for the study.

Dependent variable	Condition		Child’s age		Age × Condition		Time		Time × Condition		Time × Age	
	*F*(2,358)	η^2^	*F*(1,358)	η^2^	*F*(2,358)	η^2^	*F*(1,358)	η^2^	*F*(1,358)	η^2^	*F*(2,358)	η^2^
Meaning of Life	109.5^∗^	0.16	409.10^∗^	0.30	179.30^∗^	0.27	0.08	0.00	3.13+	0.02	0.07	0.00
Hope	68.99^∗^	0.15	202.40^∗^	0.23	97.35^∗^	0.22	0.02	0.00	1.50	0.01	1.02	0.00
Self-Esteem	29.82^∗^	0.11	75.10^∗^	0.14	29.71^∗^	0.11	33.17^∗^	0.08	5.54+	0.03	18.45^∗^	0.04
Optimism	60.14^∗^	0.12	386.29^∗^	0.38	81.63^∗^	0.16	0.04	0.00	2.04	0.01	0.23	0.00
Positive Affect	20.23^∗^	0.08	56.42^∗^	0.11	29.63^∗^	0.12	3.71	0.01	3.55+	0.02	11.89^∗^	0.03
Stress Related Growth	265.55^∗^	0.44	144.20^∗^	0.12	83.82^∗^	0.14	0.09	0.00	8.21^∗^	0.04	5.58+	0.02

^+^p < 0.05; *p < 0.01.

The results of ANOVA demonstrated a significant main effect of the study condition on all of the dependent variables. Narrating mothers scored higher than the mothers in the other study conditions (after placebo and after the no-task condition). The results of the *post hoc* Bonferoni test indicated that mothers in all three study conditions differed significantly. The effects of the child’s age were also significant. Mothers of older children scored higher than mothers of younger children. The interaction effects of the condition and the child’s age were also significant. The differences between narrative, placebo and control groups were higher among mothers with older children than among mothers with younger children both for Time 1 and Time 2. The results of the *post hoc* Bonferoni test indicated that the mothers of younger children did not differ significantly across all three experimental conditions for Time 1 and Time 2 or the differences were marginally significant.

The main effects of time and the interaction effects between time and child’s age and time and condition were either not significant or only marginally significant. Their effect sizes were also lower in comparison to the main effects of the condition, child’s age and their interactions.

### Meaning of Life

**FIGURE 1:**
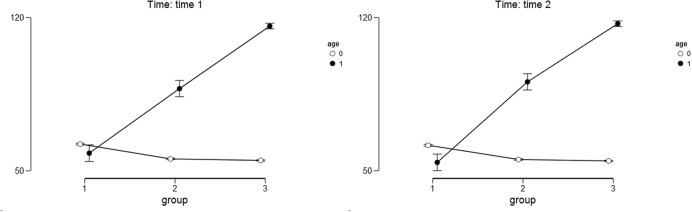
**Descriptive statistics for meaning of life as the outcome variable.** Age – 0 stands for younger children and 1 stands for older children. Group – 1 stands for control group; 2 stands for placebo group; 3 stands for narrative group. Error bars represent the 95% confidence interval (CI) of a mean.

For the meaning of life as the outcome variable (measured with PIL) we found a significant main effect of the study condition [*F*(2,358) = 109.50; *p* < 0.001; η^2^ = 0.163]. Narrating mothers scored higher on the PIL Scale (*M*_T1_ = 85.16; *SD*_T1_ = 32.40; *M*_T2_ = 85.59; *SD*_T2_ = 32.85) than the mothers in the other study conditions: after placebo (*M*_T1_ = 71.07; *SD*_T1_ = 22.95; *M*_T2_ = 72.45; *SD*_T2_ = 23.46) and after the no-task condition (*M*_T1_ = 60.06; *SD*_T1_ = 20.48; *M*_T2_ = 57.60; *SD*_T2_ = 18.07). The results of the *post hoc* Bonferoni test indicated that mothers in all three study conditions differed significantly from one another (*p* < 0.05). The main effect of child’s age was also significant [*F*(1,358) = 409.10; *p* < 0.001; η^2^ = 0.304]. Mothers of older children scored higher on the PIL Scale (*M*_T1_ = 86.89; *SD*_T1_ = 28.73; *M*_T2_ = 86.79; *SD*_T2_ = 29.52) than the mothers of younger children (*M*_T1_ = 57.38; *SD*_T1_ = 16.80; *M*_T2_ = 57.03; *SD*_T2_ = 15.91). The interaction effect of the study condition and child’s age was also significant [*F*(2,358) = 179.30; *p* < 0.001; η^2^ = 0.267]. The differences between narrative, placebo, and control groups were higher among mothers with older children than among mothers with younger children both at Time 1 and Time 2 (see **Table 2** and **Figure 1** for details). The results of the *post hoc* Bonferoni test indicated that among mothers of younger children only the control and narrative groups differed significantly.

**Table 2:** Descriptive statistics for meaning of life as the outcome variable.

		Time 1			Time 2		
		*M*	*SD*	*N*	*M*	*SD*	*N*
Younger children	Control	62.20	21.098	59	61.64	19.887	59
	Placebo	55.43	15.624	61	55.15	14.753	61
	Narrative	54.73	11.877	62	54.50	11.432	62
Older children	Control	58.05	19.835	63	53.81	15.390	63
	Placebo	87.52	17.222	58	90.64	15.919	58
	Narrative	116.10	7.799	61	117.20	6.838	61

The main effect of time was not significant [*F*(1,358) = 0.078; *p* = 0.78; η^2^ = 0.000], nor were the interactions between time and child’s age [*F*(1,358) = 0.073; *p* = 0.79; η^2^ = 0.000]; time, condition and child’s age [*F*(2,358) = 2.63; *p* = 0.07; η^2^ = 0.000]. Only the interaction between time and condition was marginally significant [*F*(2,358) = 3.128; *p* < 0.05; η^2^ = 0.014].

### Hope

**FIGURE 2:**
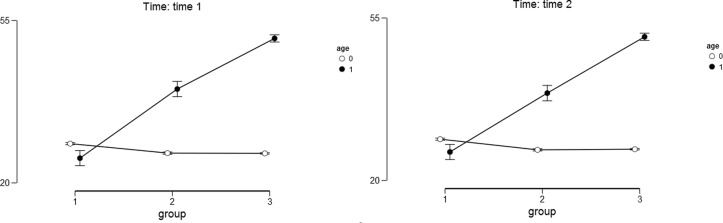
**Descriptive statistics for hope as the outcome variable.** Age – 0 stands for younger children and 1 stands for older children. Group – 1 stands for control group; 2 stands for placebo group; 3 stands for narrative group. Error bars represent the 95% CI of a mean.

For the hope as the outcome variable we found a significant main effect of the study condition [*F*(2,358) = 68.99; *p* < 0.0001; η^2^ = 0.154]. Narrating mothers scored higher on the Hope Scale (*M*_T1_ = 38.65; *SD*_T1_ = 13.90; *M*_T2_ = 38.75; *SD*_T2_ = 13.83) than the mothers in the other study conditions: after placebo (*M*_T1_ = 33.15; *SD*_T1_ = 11.10; *M*_T2_ = 32.656; *SD*_T2_ = 10.96) and after the no-task condition (*M*_T1_ = 26.84; *SD*_T1_ = 9.51; *M*_T2_ = 27.48; *SD*_T2_ = 8.69). The results of the *post hoc* Bonferoni test indicated that mothers in all three study conditions differed significantly from one another (*p* < 0.05). The main effect of child’s age was also significant [*F*(1,358) = 202.40; *p* < 0.001; η^2^ = 0.227]. Mothers of older children scored higher on the Hope Scale (*M*_T1_ = 38.73; *SD*_T1_ = 12.89; *M*_T2_ = 38.50; *SD*_T2_ = 12.68) than mothers of younger children (*M*_T1_ = 27.07; *SD*_T1_ = 9.15; *M*_T2_ = 27.40; *SD*_T2_ = 8,84). The interaction effect of the study condition and child’s age was also significant [*F*(2,358) = 97.35; *p* < 0.001; η^2^ = 0.218]. The differences between the narrative, placebo, and control groups were higher and significant only among mothers with older children both for Time 1 and Time 2 (see **Table 3** and **Figure 2** for details). The results of the *post hoc* Bonferoni test indicated that the mothers of younger children in all three study conditions did not differed significantly at Time 1 and Time 2.

**Table 3:** Descriptive statistics for hope as the outcome variable.

		Time 1			Time 2		
		*M*	*SD*	*N*	*M*	*SD*	*N*
Younger children	Control	28.46	9.884	59	28.90	9.523	59
	Placebo	26.43	9.523	61	26.61	9.072	61
	Narrative	26.37	7.964	62	26.74	7.834	62
Older children	Control	25.33	8.964	63	26.14	7.664	63
	Placebo	40.22	7.764	58	38.83	9.181	58
	Narrative	51.13	3.739	61	50.95	5.091	61

The main effect of time was not significant [*F*(1,358) = 0.017, *p* = 0.90, η^2^ = 0.000] and neither were the interactions between time and child’s age [*F*(1,358) = 1.018, *p* = 0.31, η^2^ = 0.003], time and study condition [*F*(2,358) = 1.498, *p* = 0.225, η^2^ = 0.008], and time, study condition, and child’s age [*F*(2,358) = 0.927, *p* = 0.40, η^2^ = 0.005].

### Self-Esteem

**FIGURE 3:**
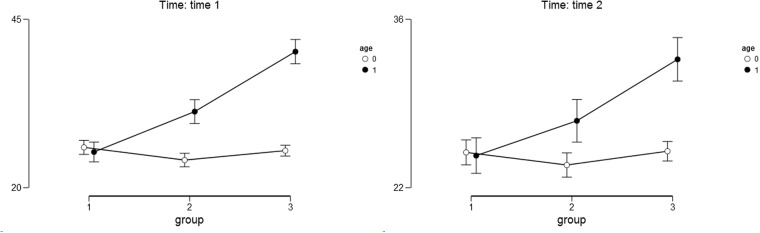
**Descriptive statistics for self-esteem as the outcome variable.** Age – 0 stands for younger children and 1 stands for older children. Group – 1 stands for control group; 2 stands for placebo group; 3 stands for narrative group. Error bars represent the 95% CI of a mean.

For the self-esteem as the outcome variable we found a significant main effect of the study condition [*F*(2,357) = 29.82, *p* < 0.001, η^2^ = 0.108]. Narrating mothers scored higher on the Self-Esteem Scale (*M*_T1_ = 32.79, *SD*_T1_ = 8.90 *M*_T2_ = 28.82, *SD*_T2_ = 8.94) than the mothers in the remaining study conditions: after placebo (*M*_T1_ = 27.58, *SD*_T1_ = 7.91; *M*_T2_ = 25.66, *SD*_T2_ = 7.18), and after the no-task condition (*M*_T1_ = 25.63, *SD*_T1_ = 7.79; *M*_T2_ = 24.80, *SD*_T2_ = 5.94). The results of the *post hoc* Bonferoni test indicated that groups of mothers in all three study conditions differed significantly from one another (*p* < 0.05). The main effect of child’s age was also significant [*F*(1,357) = 75.10, *p* < 0.001, η^2^ = 0.136]. Mothers of older children scored higher on the Self-Esteem Scale (*M*_T1_ = 32.21, *SD*_T1_ = 9.08; *M*_T2_ = 28.28, *SD*_T2_ = 9.20) compared to the mothers of younger children (*M*_T1_ = 25.19, *SD*_T1_ = 7.19; *M*_T2_ = 24.62, *SD*_T2_ = 5.09). The interaction effect of the study condition and child’s age was also significant [*F*(2,357) = 29.71, *p* < 0.001, η^2^ = 0.108]. The differences between narrative, placebo and control groups were higher and statistically significant only among mothers with older children both at Time 1 and Time 2 (see **Table 4** and **Figure 3** for details). The results of the *post hoc* Bonferoni test indicated that the mothers of younger children in all three study conditions did not differ significantly neither at Time 1 nor at Time 2.

**Table 4:** Descriptive statistics for self-esteem as the outcome variable.

		Time 1			Time 2		
		*M*	*SD*	*N*	*M*	*SD*	*N*
Younger children	Control	25.98	7.583	59	24.93	5.139	59
	Placebo	24.10	7.469	61	23.89	4.940	61
	Narrative	25.50	6.483	62	25.03	5.185	62
Older children	Control	25.30	8.023	63	24.67	6.636	63
	Placebo	31.30	6.603	57	27.56	8.627	57
	Narrative	40.20	4.956	61	32.67	10.251	61

Although the main effect of time [*F*(1,357) = 33.167, *p* < 0.001, η^2^ = 0.076] and the interactions between time and child’s age [*F*(1,358) = 18.451, *p* < 0.001, η^2^ = 0.042] time and study condition [*F*(2,357) = 5.540, *p* < 0.01, η^2^ = 0.025], and time, study condition, and child’s age [*F*(2,357) = 7.582, *p* < 0.001, η^2^ = 0.035] were also significant, the effect sizes were considerably lower in comparison to the main effects of the study condition, child’s age and their interactions.

### Optimism

**FIGURE 4:**
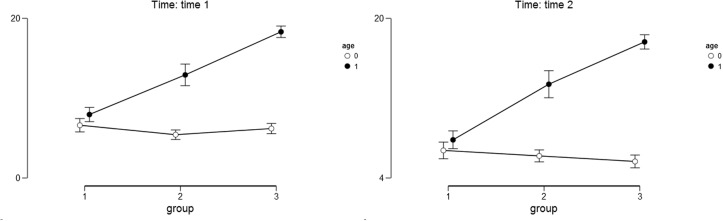
**Descriptive statistics for optimism as the outcome variable.** Age – 0 stands for younger children and 1 stands for older children. Group – 1 stands for control group; 2 stands for placebo group; 3 stands for narrative group. Error bars represent the 95% CI of a mean.

**FIGURE 5:**
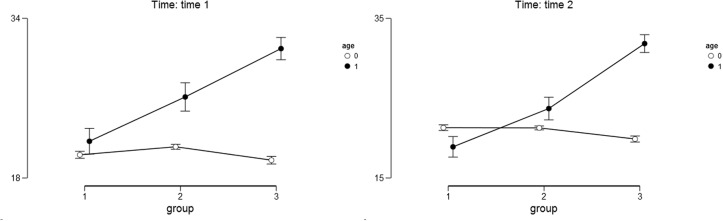
**Descriptive statistics for positive affect as the outcome variable.** Age – 0 stands for younger children and 1 stands for older children. Group – 1 stands for control group; 2 stands for placebo group; 3 stands for narrative group. Error bars represent the 95% CI of a mean.

For the optimism as the outcome variable we found a significant main effect of the study condition [*F*(2,358) = 60.14, *p* < 0.001, η^2^ = 0.117]. Narrating mothers scored higher on the Optimism Scale (*M*_T1_ = 12.20, *SD*_T1_ = 6.95; *M*_T2_ = 11.60, *SD*_T2_ = 6.67) than the mothers in the other study conditions: after placebo (*M*_T1_ = 9.08, *SD*_T1_ = 6.07; *M*_T2_ = 9.71, *SD*_T2_ = 6.01) and after the no-task condition (*M*_T1_ = 7.30, *SD*_T1_ = 4.22; *M*_T2_ = 7.21, *SD*_T2_ = 4.20). The results of the *post hoc* Bonferoni test indicated that groups of mothers in all three study conditions differed significantly from one another (*p* < 0.05). The main effect of child’s age was also significant [*F*(1,358) = 386.29, *p* < 0.001, η^2^ = 0.376]. Mothers of older children scored higher on the LOT-R scale (*M*_T1_ = 13.01, *SD*_T1_ = 6.38; *M*_T2_ = 12.89, *SD*_T2_ = 6.16) than the mothers of younger children (*M*_T1_ = 6.07, *SD*_T1_ = 3.44; *M*_T2_ = 6.20, *SD*_T2_ = 3.39). The interaction effect of the study condition and child’s age was also significant [*F*(2,358) = 81.63, *p* < 0.001, η^2^ = 0.159]. The differences between the narrative, placebo, and control groups were higher among mothers with older children than among mothers with younger children both at Time 1 and Time 2 (see **Table 5** and **Figures 4** and **5** for details). The results of the *post hoc* Bonferoni test indicated that the mothers of younger children in all three study conditions did not differ significantly neither at Time 1 nor at Time 2 and the between-groups differences were only marginally significant at Time 1.

**Table 5:** Descriptive statistics for optimism as the outcome variable.

		Time 1			Time 2		
		*M*	*SD*	*N*	*M*	*SD*	*N*
Younger children	Control	6.610	3.913	59	6.763	3.607	59
	Placebo	5.426	2.889	61	6.213	3.292	61
	Narrative	6.194	3.416	62	5.661	3.073	62
Older children	Control	7.952	4.427	63	7.825	4.665	63
	Placebo	12.914	6.179	58	13.397	6.041	58
	Narrative	18.311	3.319	61	17.639	2.714	61

The main effect of time was not significant [*F*(1,358) = 0.004, *p* = 0.95, η^2^ = 0.000] and neither were the interactions between time and child’s age [*F*(1,358) = 0.233, *p* = 0.63, η^2^ = 0.001], time and study condition [*F*(2,358) = 2.035, *p* = 0.13, η^2^ = 0.011], and time, study condition and child’s age [*F*(2,358) = 0.011, *p* = 0.99, η^2^ = 0.000].

### Positive Affect

For the positive affect as the outcome variable we found a significant main effect of the study condition [*F*(2,357) = 20.23, *p* < 0.001, η^2^ = 0.079]. Narrating mothers scored higher on the Positive Affect Scale (*M*_T1_ = 25.33, *SD*_T1_ = 8.45; *M*_T2_ = 25.81, *SD*_T2_ = 8.29) than the mothers in the other study conditions: after placebo (*M*_T1_ = 23.54, *SD*_T1_ = 8.12; *M*_T2_ = 22.45, *SD*_T2_ = 7.65) and after the no-task condition (*M*_T1_ = 21.02, *SD*_T1_ = 7.23; *M*_T2_ = 20.07, *SD*_T2_ = 5.59). The results of the *post hoc* Bonferoni test indicated that groups of mothers in all three study conditions differed significantly from one another (*p* < 0.05). The main effect of child’s age was also significant [*F*(1,357) = 56.42, *p* < 0.001, η^2^ = 0.110]. Mothers of older children scored higher on the Positive Affect Scale (*M*_T1_ = 26.21, *SD*_T1_ = 7.73; *M*_T2_ = 24.77, *SD*_T2_ = 8.06) than the mothers of younger children (*M*_T1_ = 20.41, *SD*_T1_ = 7.47; *M*_T2_ = 20.81, *SD*_T2_ = 6.63). The interaction effect of the study condition and child’s age was also significant [*F*(2,357) = 29.63, *p* < 0.001, η^2^ = 0.115]. The differences between the narrative, placebo, and control groups were higher and statistically significant only among the mothers with older children both at Time 1 and Time 2 (see **Table 6** and **Figure 5** for details). The results of the *post hoc* Bonferoni test indicated that the mothers of younger children in all three study conditions did not differ significantly neither at Time 1 nor at Time 2.

**Table 6:** Descriptive statistics for PANAS as the outcome variable.

		Time 1			Time 2		
		*M*	*SD*	*N*	*M*	*SD*	*N*
Younger children	Control	20.32	7.533	59	21.31	6.610	59
	Placebo	21.13	8.111	61	21.28	7.356	61
	Narrative	19.79	6.792	62	19.89	5.854	62
Older children	Control	21.68	6.925	63	18.90	4.165	63
	Placebo	26.12	7.356	57	23.70	7.822	57
	Narrative	30.97	5.868	61	31.84	5.643	61

Although the interactions between time and child’s age [*F*(1,357) = 11.890, *p* < 0.001, η^2^ = 0.030], time and study condition [*F*(2, 357) = 3.551, *p* < 0.05, η^2^ = 0.018], time, study condition and child’s age [*F*(2,357) = 6.457, *p* < 0.01, η^2^ = 0.033] were statistically significant, the effect sizes were considerably lower in comparison to the main effects of study condition, child’s age and their interactions. The main effect of time was only marginally significant [*F*(1,357) = 3.706, *p* = 0.06, η^2^ = 0.009].

### Stress Related Growth

**FIGURE 6:**
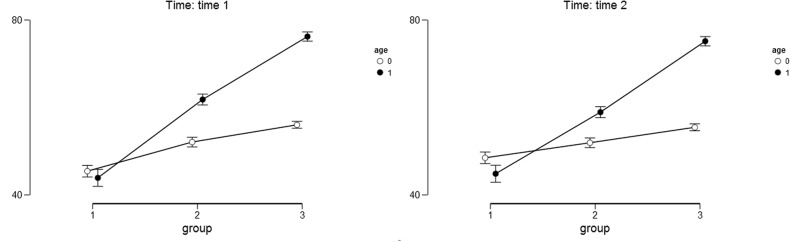
**Descriptive statistics for the stress-related growth as the outcome variable.** Age – 0 stands for younger children and 1 stands for older children. Group – 1 stands for control group; 2 stands for placebo group; 3 stands for narrative group. Error bars represent the 95% CI of a mean.

For the Stress Related Growth Scale as the outcome variable we found a significant main effect of the study condition [*F*(2,358) = 265.55, *p* < 0.001, η^2^ = 0.44]. Narrating mothers scored higher on the Stress Related Growth Scale (*M*_T1_ = 66.05, *SD*_T1_ = 11.45; *M*_T2_ = 65.24, *SD*_T2_ = 11.027) than the mothers in the other study conditions: after placebo (*M*_T1_ = 56.84, *SD*_T1_ = 9.06; *M*_T2_ = 55.34, *SD*_T2_ = 7.91) and after the no-task condition (*M*_T1_ = 44.65, *SD*_T1_ = 10.05; *M*_T2_ = 46.61, *SD*_T2_ = 9.23). The results of the *post hoc* Bonferoni test indicated that the groups of mothers in all three study conditions differed significantly from one another (*p* < 0.05). The main effect of child’s age was also significant [*F*(1,358) = 144.20, *p* < 0.001, η^2^ = 0.12]. Mothers of older children scored higher on the Stress Related Growth Scale (*M*_T1_ = 60.45, *SD*_T1_ = 15.36; *M*_T2_ = 59.50, *SD*_T2_ = 14.49) than the mothers of younger children (*M*_T1_ = 51.28, *SD*_T1_ = 9.68; *M*_T2_ = 51.02, *SD*_T2_ = 7.68). The interaction effect of the study condition and child’s age was also significant [*F*(2, 358) = 83.82, *p* < 0.001, η^2^ = 0.14]. The differences between the narrative, placebo and control groups were higher among mothers with older children than among mothers with younger children both at Time 1 and Time 2 (see **Table 7** and **Figure 6** for details).

**Table 7:** Descriptive statistics for stress-related growth as the outcome variable.

		Time 1			Time 2		
		*M*	*SD*	*N*	*M*	*SD*	*N*
Younger children	Control	45.44	10.669	59	48.49	7.877	59
	Placebo	52.10	9.609	61	51.93	7.795	61
	Narrative	56.03	4.722	62	55.47	5.659	62
Older children	Control	43.90	9.461	63	44.86	10.088	63
	Placebo	61.83	6.671	58	58.93	6.313	58
	Narrative	76.23	5.897	61	75.16	3.984	61

The main effect of time was not significant [*F*(1,358) = 0.093, *p* = 0.76, η^2^ = 0.00]. The interaction between time and child’s age was only marginally significant [*F*(1,358) = 5.583, *p* = 0.02, η^2^ = 0.015] as was the interaction between time and condition [*F*(1,358) = 8.206, *p* < 0.001, η^2^ = 0.04]. The interaction between time, study condition and child’s age was not significant [*F*(2,358) = 0.781, *p* = 0.46, η^2^ = 0.004].

### Mediations

In line with the theoretical argument we assumed that the effect of the intervention on self-esteem, hope, positive emotions, optimism, and growth would be mediated via the increase in the sense of meaning of life. Therefore, we conducted five mediation analyses separately for each outcome variable. We limited the analyses to the group of mothers with older children because the results of the ANOVAs demonstrated that there were no significant effects of the experimental manipulation among the mothers of younger children. The zero-order correlations between the variables can be found in the Supplementary Materials for the article. The analyses were performed on the data at the first time point (Time 1) but he same pattern of results was found also for the second time point (Time 2).

**FIGURE 7:**
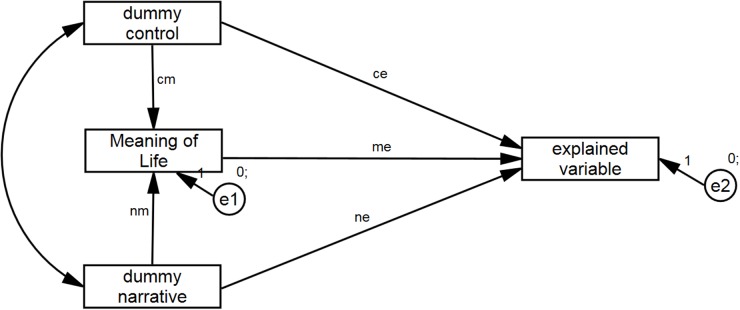
**General mediation model**.

The condition variable (control vs placebo vs narrative group) was recoded into two dummy variables: control and narrative. The reference group for the dummy was the placebo group. The general model for the mediation is presented in **Figure 7**. Standardized regression coefficients for each model are presented in **Table 8**. The results are highly consistent. In each case a partial mediation was found. The effect of the manipulation on the outcome variables was mediated via the increase in the sense of meaning of life.

**Table 8:** Standardized regression coefficients for mediation model.

Outcome variable	cm^a^	nm^a^	ce^a^	me^a^	ne^a^	Cm × me (Bootstrap BCI intervals)^b^	nm × me (Bootstrap BCI intervals)^b^	*R*^2^
Growth	-0.49^∗∗^	0.47^∗∗^	-0.40^∗∗^	0.32^∗∗^	0.29^∗∗^	-0.16^∗∗^ [-0.26; -0.08]	0.15^∗∗^ [0.08;0.23]	0.79
Optimism	-0.049^∗∗^	0.47^∗∗^	-0.19^∗^	0.36^∗∗^	0.23^∗∗^	-0.18^∗∗^ [-0.30; -0.07]	0.17^∗∗^ [0.06;0.30]	0.49
Self-Esteem	-0.049^∗∗^	0.47^∗∗^	-0.13	0.40^∗∗^	0.26^∗∗^	-0.20^∗∗^ [-0.32; -0.09]	0.19^∗∗^ [0.08;0.30]	0.51
Hope	-0.49^∗∗^	0.47^∗∗^	-0.38^∗∗^	0.34^∗∗^	0.24^∗∗^	-0.17^∗∗^ [-0.29; -0.07]	0.16^∗∗^ [0.07;0.27]	0.73
Positive Affect	-0.49^∗∗^	0.47^∗∗^	-0.02	0.54^∗∗^	0.03	-0.27^∗∗^ [-0.039; -0.15]	0.26^∗∗^ [0.14;0.37]	0.34

*^∗^p < 0.05; ^∗∗^p < 0.001; ^a^path names as depicted in **Figure 7**; ^b^estimation of indirect (mediated) effects. The numbers in parenthesis denote lower and upper levels of bootstrap bias corrected confidence intervals with 95% confidence level (number of bootstrap = 2500). As proposed by Preacher and Hayes (2004) the indirect effect is significant when the BCI intervals do not encompass 0.*

### Level of Narrative Coherence of the Text Content

To test assumption that mothers of younger children do not have sufficient psychological resources to construct meaningful coherent stories about their motherhood we compared the narrative coherence of their texts with text of older children’s mothers. The one-way ANOVA results confirmed the hypotheses. Narrative coherence was lower for mothers of younger children (*M* = 3.06; *SD* = 0.93) than for mothers of older children (*M* = 5.13; *SD* = 1.17), *F*(1,121) = 118.62, *p* < 0.001, η^2^ = 0.50. As expected, the level of narrative coherence of the text was positively related to well-being after the writing. All 0 order correlations between the narrative coherence and indicators of well-being were significantly positive for both time 1 and time 2 measure/*p* < 0.001, The correlations for time 1 are presented in the first place/: Meaning 0.69^∗^/0.67^∗^; Hope 0.64^∗^/0.63^∗^; Self_Esteem 0.56^∗^/0.29^∗^; Optimism 0.62^∗^/0.62^∗^; Positive Affect 0.46^∗^/0.49^∗^; Stress Related Growth 0.58^∗^/0.63^∗^

## Discussion

Being a mother of a child with autism spectrum disorders is a life-long challenge. In order to cope positively with this challenge, mothers must find a meaning of their role in this situation. Our expectations were that the self-reflections, triggered when a mother were construing, re-construing or elaborating the story of her motherhood, would help her with this life-task. The findings indicate that the narrative construction of difficult motherhood by mothers of older children with autism does indeed result in several positive outcomes and that these outcomes are interrelated. The data suggest that, in case of these mothers, construing a narrative results predominantly in an increase in their meaning of life. This initiates a sequence of other important changes which together form an integrated psychological process in which a growing understanding of the situation, their role in it and its place in their life contribute to an increase in self-esteem and hope for success in coping with troubles and tasks. All these effects contribute to the presence of more balanced and more positive emotions related to the complicated motherhood.

We may assume, that for mothers of children with ASD, developing or elaborating narrative identity provides a meaningful frame helping them understand their wishes, desires, and fears. Subsequently, the mothers become more determined to commit and carry out the tasks as well as immunized against conflicts, feelings of helplessness, and exhaustion. They become more explicit and verbalized as moving forces within a story context. Self-narrative equips the woman with better cognitive control over the ongoing events as well as those envisioned in the future. Hope for successful coping leads to optimism and to more positive emotional balance. This way, the troubled times and challenges generate lower anxiety and uncertainty. These processes not only increase the mothers’ well-being, but also should help them to care better for the child.

The results suggest, however, that the above processes occur only under certain conditions. We assume that successful narrative meaning-making is possible when a person has an opportunity to think thoughtfully and creatively. An important condition for those processes to occur is a lack of strong negative emotions, in this case - emotions of uncertainty and anxiety. What distinguishes mothers of younger children just diagnosed as having ASD, from mothers of older children with ASD is the unexpectedness and novelty of the situation of being a mother to a child with autism. We assumed that higher uncertainty and anxiety that accompany this situation disorganize the complex cognitive processes that are crucial for a successful construction of a personal story that integrates different aspects of a challenge and its place in one’s present life. The assumption was supported: Mothers of younger children asked to write stories have produced text with lower level of narrative coherency in comparison to text by mothers of older children. The accompanying results indicate that the level of text narrative coherency is positively related to the magnitude of impact of the narrative condition. It means that the effects of narrative interpretation of own challenge on well-being and resources for coping, depend on situational and personal opportunities to think and to create an integrated and therefore meaningful story. Specifically, when a person is too close to a beginning of a dramatic change in life, strong anxiety-based emotions may block narrative reflection and construction of such story.

The research conducted by Duarte et al. (2005) showed a relationship between the age of the children with ASD and the strength of stress experienced by their parents: Mothers of younger children have a higher level of stress than mothers of older children. This can be attributed to the burden of the diagnosis, searching for help, and for effective means to deal with the challenges. The differences in the results obtained in our study of mothers of older children (9–12 years) and younger children (1–3 years) can be explained by the parents’ emotional reaction to the expected loss of a healthy child (Siegel, 1997). In one of the models of this process, presented by Bristor (1984) six phases of parents’ experience were identified. They are adaptive and help with overcoming the feeling of loss, guilt and other negative emotions associated with parenting a child with ASD.

Empowerment is an important factor in the context of dealing with the challenges of motherhood form mothers of children with ASD (Pisula, 2007). The surveyed mothers of older children have already gained a lot of experience with childcare and that has strengthened their sense of parental competence. The surveyed mothers of younger children received their child’s diagnosis recently and their experiences belong to the first phase of adaptation. During this time, parents experience shock after receiving information about child developmental disorders and find it difficult to recognize what actually happened; they experience a sense of confusion, panic, and negatively rate their capacity to cope with childcare. No outward signs of their children’s development difficulties is the reason that their different behavior is sometimes considered as “bad manners” (Pakenham et al., 2005; Portway and Johnson, 2005) and provokes critical remarks about their mothers. In Poland still the sole responsibility for raising a child is attributed to the mothers.

In the initial phases of the adaptation process parents of child with ASD attempt to overcome losses by using two coping strategies: they attempt to maintain the status quo (e.g., by rejecting the diagnosis of ASD, searching for another specialist, collecting evidence that contradicts the diagnosis) or they distance themselves from the object of their worry (Pisula, 2007). With time and through the process of adaptation to the challenges related to bringing up a child with ASD, parental well-being improves. This conclusion was supported by the results of a study conducted by Gray (2002) in which the parents of children with autism were surveyed twice with an interval of 8–10 years. During the second measurement, parents declared a better mood than during the first measurement, less health problems, and lower stress levels. King et al. (2000) also found that parents’ situation improved over time. Their values system and goals changed, which enabled them to positively adapt to the requirements. The surveyed mothers of older children already had the time to fully recognize the situation, assess the amount of the necessary changes, develop ways of coping with the challenges and make changes (often positive) taking into account new circumstances.

The presented conclusions need more direct empirical support. The results indicate that narrative writing is associated with a specific pattern of outcomes. This pattern suggests that there is an underlying process that creates and maintains a meaningful story framework when someone copes with a difficult challenge. These results indirectly support the claim that the narrative understanding and the development of a narrative identity were the main factors in the observed effects. However, there is a need for more straightforward evidences that the narrative construction is a specific and dominant factor in these processes. One possibility is to observe the relationships between content characteristics of self-narratives (e.g., clarity and other characteristics of the story plot; McAdams, 2006; McAdams et al., 2006) and the expected outcomes. However, this requires content analyses of much more elaborated self-stories than those obtained in our research. Another limitation of the study is the lack of more direct observation of the interplay between mother’s self-narrative processes and changes in her attitudes to the child as well as coping and caring with the child, especially while the child grows up. Diaries method may by useful to observe these relationships.

Presented data provides some support for the sustainability of the narrative effects. Even a few days of narrative mindfulness can become days when a life story is created and begins to influence the person. In such case the story is still unfinished and the mother and the family may continue to develop it in line with the incoming problems, for example those related to the child growing up. An important condition for the story’s durability is the communication and cooperation between partners in the family: the developing story should be shared: negotiated and maintained within the family (Lalvani, 2011; Lalvani and Polvere, 2013). Within this social context the story begins to shape the identities of the mother, the child, and other family members and thus influences their attitudes, emotions, and behavior. The role of a family in maintaining and enacting a new self-narrative story, or coordinated self-stories, is an interesting problem for further research.

## Ethics Statement

The study was approved by the ethics Committee in Maria Grzegorzewska University, Warsaw, Poland. The participants were informed about the detailed procedure of the study. Before participating in the main study they had to sign the consent form and were informed that the participation in the study is voluntary and that it is possible to leave the study in any time. It was also emphasized that refusal to participate will not result in any consequences.

## Author Contibutions

JT: Theory and hypothesis; conceived and designed the experiments; contributed reagents/materials/analysis tools; prepared the manuscript. AWR: Hypothesis; conceived and designed the experiments; contributed reagents/materials/analysis tools; performed the experiments; prepared the manuscript. ADW: Prepared analysis tools; analyzed the data; prepared the manuscript (the Results section)

## Conflict of Interest Statement

The authors declare that the research was conducted in the absence of any commercial or financial relationships that could be construed as a potential conflict of interest.

## References

[B1] American Psychiatric Association [APA] (2013). *Diagnostic and Statistical Manual of Mental Disorders*, 5th Edn. Washington, DC: American Psychiatric Publishing.

[B2] Armeli S. Gunthert K. Cohen L. (2001). Stressor appraisal, coping, and post–event outcomes: the dimensionality and antecedents of stress–related growth. *J. Soc. Clin. Psychol.* 20 366–395. 10.1521/jscp.20.3.366.22304

[B3] Baker J. K. Seltzer M. M. Greenberg J. S. (2011). Longitudinal effects of adaptability on behavior problems and maternal depression in families of adolescents with autism. *J. Fam. Psychol.* 25 601–609. 10.1037/a0024409 21668120 PMC3987806

[B4] Baker-Ericzén M. J. Brookman–Frazee L. Stahmer A. (2005). Stress levels and adaptability in parents of toddlers with and without autism spectrum disorders. *Res. Pract. Persons Severe Disabil.* 30 194–204. 10.2511/rpsd.30.4.194

[B5] Barnett D. Clements M. Kaplan–Estrin M. Fialka J. (2003). Building new dreams supporting parents’ adaptation to their child with special needs. *Infants Young Child.* 16 184–200. 10.1097/00001163-200307000-00002

[B6] Baumeister R. F. Newman L. S. (1994). How stories make sense of personal experience: motives that shape autobiographical narratives. *Pers. Soc. Psychol. Bull.* 20 676–690. 10.1177/0146167294206006

[B7] Bishop S. L. Richler J. Cain A. C. Lord C. (2007). Predictors of perceived negative impact in mothers of children with autism spectrum disorders. *Am. J. Ment. Retard.* 112 450–461. 10.1352/0895-8017(2007)112[450:POPNII]2.0.CO;2 17963436

[B8] Brewer W. F. Lichtenstein E. H. (1981). “Event schemas, story schemas, and story grammars,” in *Attention and Performance IX*, eds Long J. Baddeley A. (Hillsdale, NJ: Erlbaum), 363–379.

[B9] Bristor M. W. (1984). The birth of a handicapped child: a wholistic model for grieving. *Fam. Relat.* 33 25–32. 10.2307/584586

[B10] Brown I. Brown R. (2003). *Quality of Life and Disability: An Approach for Community Practitioners.* London: Jessica Kingsley Publishers.

[B11] Bruner J. (1990). *Acts of Meaning: Four lectures on Mind and Culture.* Cambridge, MA: Harvard University Press.

[B12] Burton C. M. King L. A. (2008). Effects of (very) brief writing on health: the two-minute miracle. *Br. J. Health Psychol.* 13 9–14. 10.1348/135910707X250910 18230223

[B13] Clarke P. MacLeod C. (2013). “The impact of anxiety on cognitive task performance,” in *Secondary Influences on Neuropsychological Test Performance: Research Findings and Practical Applications*, ed. Arnett Peter A. (New York, NY: Oxford University Press), 93–116.

[B14] Crumbaugh J. Maholick L. (1964). An experimental study of existentialism: the psychometric approach to Frankl’s concept of noogenic neurosis. *J. Clin. Psychol.* 20 200–207. 10.1002/1097-4679(196404)20:2<200::AID-JCLP2270200203>3.0.CO;2-U 14138376

[B15] Davis N. Carter A. (2008). Parenting stress in mothers and fathers of toddlers with autism spectrum disorders: associations with child characteristics. *J. Autism. Dev. Disord.* 38 1278–1291. 10.1007/s10803-007-0512-z 18240012

[B16] Duarte C. S. Bordin I. A. Yazigi L. Mooney J. (2005). Factors associated with stress in mothers of children with autism. *Autism* 9 416–427. 10.1177/1362361305056081 16155057

[B17] Dzwonkowska I. Lachowicz-Tabaczek K. Łaguna M. (2007). Skala samooceny SES Morrisa Rosenberga – polska adaptacja metody [The Rosenberg Self-Esteem Scale – Polish adaptation]. *Psychol. Spoeczna* 2 164–176.

[B18] Emerson E. (2003). Mothers of children and adolescents with intellectual disability: social and economic situation, mental health status, and the self–assessed social and psychological impact of the child’s difficulties. *J. Intellect. Disabil. Res.* 47 385–399. 10.1046/j.1365-2788.2003.00498.x 12787168

[B19] Estes A. Munson J. Dawson G. Koehler E. Zhou X. H. Abbott R. (2009). Parenting stress and psychological functioning among mothers of preschool children with autism and developmental delay. *Autism* 13 375–387. 10.1177/1362361309105658 19535467 PMC2965631

[B20] Eysenck M. W. (2013). “The impact of anxiety on cognitive performance,” in *Cognition and Motivation: Forging an Interdisciplinary Perspective*, ed. Kreitler S. (New York, NY: Cambridge University Press), 96–108.

[B21] Fajkowska M. Marszał-Wiśniewska M. (2009). Właściwości psychometryczne Skali Pozytywnego i Negatywnego Afektu [The psychometric characteristics of the Positive and Negative Affect Schedule - Extended Version (PANAS-X). *Przegl. Psychol.* 52 355–388.

[B22] Farber B. (1968). *Mental Retardation. Its Social Contex and Social Consequences.* Boston: Houghton Miﬄin Company.

[B23] Feldman M. McDonald L. Serbin L. Stack D. Secco M. L. Yu C. T. (2007). Predictors of depressive symptoms in primary caregivers of young children with or at risk for developmental delay. *J. Intellect. Disabil. Res.* 51 609–619. 10.1111/j.1365-2788.2006.00941.x 17598874

[B24] Frattaroli J. (2006). Experimental disclosure and its moderators: a meta–analysis. *Psychol. Bull.* 6 823–865. 10.1037/0033-2909.132.6.823 17073523

[B25] Goin-Kochel R. P. Myers B. J. (2005). Congenital versus regressive onset of autism spectrum disorders: parents beliefs about causes. *Focus Autism Other Dev. Disabl.* 20 169–179. 10.1177/10883576050200030501

[B26] Graesser A. C. Singer M. Trabasso T. (1994). Constructing inferences during narrative text comprehension. *Psychol. Rev.* 101 371–395. 10.1037/0033-295X.101.3.371 7938337

[B27] Graungaard A. H. Skov L. (2007). Why do we need a diagnosis? A qualitative study of parents’ experiences, coping and needs, when the newborn child is severely disabled. *Child Care Health Dev.* 33 296–307. 10.1111/j.1365-2214.2006.00666.x 17439444

[B28] Gray D. E. (2002). Ten years old: a longitudinal study of families of children with autism. *J. Intellect. Dev. Disabil.* 27 215–222. 10.1080/1366825021000008639

[B29] Harwood K. McLean N. Durkin K. (2007). First-time mothers’ expectations of parenthood: what happens when optimistic expectations are not matched by later experiences? *Dev. Psychol.* 43 1–12. 10.1037/0012-1649.43.1.1 17201504

[B30] Hastings R. P. (2003). Child behaviour problems and partner mental health as correlates of stress in mothers and fathers of children with autism. *J. Intellect. Disabil. Res.* 47 231–237. 10.1046/j.1365-2788.2003.00485.x 12787155

[B31] Hastings R. P. Kovshoff H. Ward N. J. degli Espinosa F. Brown T. Remington B. (2005). Systems analysis of stress and positive perceptions in mothers and fathers of pre-school children with autism. *J. Autism Dev. Disord.* 35 635–644. 10.1007/s10803-005-0007-8 16177837

[B32] Hayes S. A. Watson S. L. (2013). The impact of parenting stress: a meta-analysis of studies comparing the experience of parenting stress in parents of children with and without autism spectrum disorder. *J. Autism Dev. Disord.* 43 629–642. 10.1007/s10803-012-1604-y 22790429

[B33] Herring S. Gray K. Taffe J. Tonge B. Sweeney D. Einfeld S. (2006). Behaviour and emotional problems in toddlers with pervasive developmental disorders and developmental delay; associations with parental mental health and family functioning. *J. Intellect. Disabil. Res.* 50 874–888. 10.1111/j.1365-2788.2006.00904.x 17100948

[B34] Juczyński Z. (2009). *Narzêdzia Pomiaru w Promocji i Psychologii Zdrowia [Measurements in Health Psychology].* Warszawa: Pracownia Testów Psychologicznych PTP.

[B35] King L. A. Scollon C. K. Ramsey C. Williams T. (2000). Stories of life transition: subjective well-being and ego development in parents of children with down syndrome. *J. Res. Pers.* 34 509–536. 10.1006/jrpe.2000.2285

[B36] Łaguna M. Trzebiński J. Zieba M. (2005). *Kwestionariusz Nadziei na Sukces [The Hope Scale].* Warszawa: Pracownia Testów Psychologicznych PTP.

[B37] Lalvani P. (2011). Constructing the (m)other: dominant and contested narratives on mothering a child with Down syndrome. *Narrat. Inq.* 21 276–293. 10.1075/ni.21.2.06lal

[B38] Lalvani P. Polvere L. (2013). Historical perspectives on studying families of children with disabilities: a case for critical research. *Disabil. Stud. Q.* 33 18–35. 10.18061/dsq.v33i3.3209

[B39] Larson E. (1998). Reframing the meaning of disability to families: the embrace of paradox. *Soc. Sci. Med.* 47 865–875. 10.1016/S0277-9536(98)00113-0 9722107

[B40] Lehnert W. G. (1981). Plot units and narrative summarization. *Cogn. Sci.* 5 293–331. 10.1207/s15516709cog0504_1

[B41] Ljubomirski S. Sousa L. Dickerhoof R. (2006). The costs and benefits of writing, talking, and thinking about life’s triumphs and defeats. *J. Pers. Soc. Psychol.* 90 692–708. 10.1037/0022-3514.90.4.692 16649864

[B42] Lopez S. J. Ciarlelli R. Coffman L. Stone M. Wyatt L. (2000). “Diagnosing for strengths: On measuring hope building blocks,” in *Handbook of Hope: Theory, Measures, and Interventions*, ed. Snyder C. R. (San Diego, CA: Academic Press), 57–85.

[B43] Marcus L. M. Kunce L. J. Schopler E. (2005). “Working with families,” in *Handbook of Autism and Pervasive Developmental Disorders*, eds Volkmar F. Paul R. Klin A. Cohen D. J. (Hoboken, NJ: Wiley), 1055–1086.

[B44] McAdams D. P. (2006). *The Redemptive Self: Stories Americans Live by.* New York, NY: Oxford University Press.

[B45] McAdams D. P. Josselson R. Lieblich A. (2006). *Identity and Story: Creating Self in Narrative.* Washington, DC: American Psychological Association.

[B46] Niederhoffer K. G. Pennebaker J. W. (2009). “Sharing one’s story: On the benefits of writing or talking about emotional experience,” in *Oxford Handbook of Positive Psychology*, eds Lopez S. J. Snyder C. R. (New York, NY: Oxford University Press), 621–632.

[B47] Pakenham K. I. Samios C. Sofronoff K. (2005). Adjustment in mothers of children with Asperger syndrome: an application of the double ABCX model of family adjustment. *Autism* 9 191–212. 10.1177/1362361305049033 15857862

[B48] Pals J. L. (2006). Narrative identity processing of difficult life experiences: pathways of personality development and positive self-transformation in adulthood. *J. Pers.* 74 1079–1110. 10.1111/j.1467-6494.2006.00403.x 16787429

[B49] Park C. L. Cohen L. H. Murch R. L. (1996). Assessment and prediction of stress-related growth. *J. Pers.* 64 71–105. 10.1111/j.1467-6494.1996.tb00815.x 8656319

[B50] Pennebaker J. W. (1993). Putting stress into words: health, linguistic and therapeutic implications. *Behav. Res. Ther.* 31 539–548. 10.1016/0005-7967(93)90105-4 8347112

[B51] Pennebaker J. W. Seagal J. (1999). Forming a story: the health benefits of narrative. *J. Clin. Psychol.* 55 1243–1254. 10.1002/(SICI)1097-4679(199910)55:10<1243::AID-JCLP6>3.0.CO;2-N 11045774

[B52] Pisula E. (2007). A comparative study of stress profi les in mothers of children with autism and those of children with down’s syndrome. *J. Appl. Res. Intellect. Disabil.* 20 274–278. 10.1111/j.1468-3148.2006.00342.x

[B53] Portway S. M. Johnson B. (2005). Do you know I have Asperger’s syndrome? Risks of a non-obvious disability. *Health Risk Soc.* 7 73–83. 10.1080/09500830500042086

[B54] Povee K. Roberts L. Bourke J. Leonard H. (2012). Family functioning in families with a child with Down syndrome: a mixed methods approach. *J. Intellect. Disabil. Res.* 56 961–973. 10.1111/j.1365-2788.2012.01561.x 22533693

[B55] Preacher K. J. Hayes A. F. (2004). SPSS and SAS procedures for estimating indirect effects in simple mediation models. *Behav. Res. Methods Instrum. Comput.* 36 717–731. 10.3758/BF03206553 15641418

[B56] Rosenberg M. (1965). *Society and the Adolescent Self-image.* New York, NY: Princeton.

[B57] Schalock R. (2004). The concept of quality of life: what we know and do not know. *J. Intellect. Disabil. Res.* 48 203–216. 10.1111/j.1365-2788.2003.00558.x 15025663

[B58] Scheier M. F. Carver C. S. Bridges M. W. (1994). Distinguishing optimism from neuroticism (and trait anxiety, self-mastery, and self-esteem): a reevaluation of the life orientation test. *J. Pers. Soc. Psychol.* 67 1063–1078. 10.1037/0022-3514.67.6.1063 7815302

[B59] Siegel B. (1997). “Coping with the diagnosis of autism,” in *Handbook of Autism and Developmental Disorders*, eds Volkmar F. Cohen D. (New York, NY: Wiley), 745–766.

[B60] Singer G. H. S. (2006). Meta–analysis of comparative studies of depression in mothers of children with and without developmental disabilities. *Am. J. Ment. Retard.* 111 155–169. 10.1352/0895-8017(2006)111[155:MOCSOD]2.0.CO;2 16597183

[B61] Singer J. A. (2004). Narrative identity and meaning making across the adult lifespan: an introduction. *J. Pers.* 72 437–460. 10.1111/j.0022-3506.2004.00268.x 15102034

[B62] Smith J. (1994). Reconstructing selves: an analysis of discrepancies between women’s contemporaneous and retrospective accounts of the transition to motherhood. *Br. J. Psychol.* 85 371–392. 10.1111/j.2044-8295.1994.tb02530.x 7921745

[B63] Smyth J. Stone A. Hurewitz A. Kaell A. (1999). Effects of writing about stressful experiences on symptom reduction in patients with asthma or rheumatoid arthritis: a randomized trial. *J. Am. Med. Assoc.* 281 1304–1309. 10.1001/jama.281.14.1304 10208146

[B64] Smyth J. True N. Souto J. (2001). Effects about writing about traumatic experiences. The necessity for narrative structuring. *J. Soc. Clin. Psychol.* 20 161–170. 10.1521/jscp.20.2.161.22266

[B65] Snyder C. R. Sympson S. C. Michael S. T. Cheavens J. (2000). “Optimism and hope constructs: variations on a positive expectancy theme,” in *Optimism and Pessimism: Implications for Theory, Research and Practice*, ed. Chang E. C. (Washington, DC: American Psychological Association), 101–123.

[B66] Trzebinski J. (1998). Self–narratives as sources of motivation. *Psychol. Lang. Commun.* 2 13–22.

[B67] van Steijn D. Oerlemans A. M. van Aken M. Buitelaar J. Rommelse N. (2014). The reciprocal relationship of ASD, ADHD, depressive symptoms and stress in parents of children with ASD and/or ADHD. *J. Autism. Dev. Disord.* 44 1064–1076. 10.1007/s10803-013-1958-9 24114582

[B68] Watson D. Clark L. A. (1999). *The PANAS-X: Manual for the Positive and Negative Affect Schedule Expanded Form.* Available at: http://ir.uiowa.edu/psychologypubs/11

[B69] Werner S. Edwards M. Baum N. Brown I. Brown R. I. Isaacs B. J. (2009). Family quality of life among families with a member who has an intellectual disability: an exploratory examination of key domains and dimensions of the revised FQOL Survey. *J. Intellect. Disabil. Res.* 53 501–511. 10.1111/j.1365-2788.2009.01164.x 19302473

[B70] Wieland N. Green S. Ellingsen R. Baker B. L. (2014). Parent–child problem solving in families of children with or without intellectual disability. *J. Intellect. Disabil. Res.* 58 17–30. 10.1111/jir.12009 23336566 PMC4861145

[B71] Zieba M. Łaguna M. Trzebiński J. (2010). Kwestionariusz zmian yciowych. [Questionnaire of Life Changes]. *Stud. Psychol.* 49 109–120.

[B72] Życińska J. Januszek M. (2011). Test Sensu Życia (Purpose in Life Test, PIL). *Czas. Psychol.* 17 133–142.

