# Supplementary material for: The Impact of Self-Narratives of Motherhood for Mothers of Children with Autism
Source: Front Psychol. 2016 Dec 5;7:1899. doi: 10.3389/fpsyg.2016.01899 (PMC5136541; doi:10.3389/fpsyg.2016.01899)
Supplement: Supplementary file 1 [file Data_Sheet_1.docx]

Supplementary Material

The impact of self–narratives of motherhood for mothers of children with autism

**Jerzy Trzebinski*, Agnieszka Wołowicz-Ruszkowska, Adrian Dominik Wójcik**

**Correspondence:** Jerzy Trzebiński: jtrzebin@gmail.com

# Supplementary Table

**Table 1 | Zero–Order Correlations between dependent variables for Time 1 and Time 2.**

|  | Growth  T1/T2 | Meaning  T1/T2 | Optimism  T1/T2 | Self– Esteem  T1/T2 | Hope  T1/T2 | PANAS_Pos  T1/T2 |
| --- | --- | --- | --- | --- | --- | --- |
| Growth | - | .85*/.85* | .70*/.65* | .64*/.42* | .79*/79* | .55*/.62* |
| Meaning | -.07/-.03 | - | .67*/.76* | .69*/.40* | .80*/.84* | .58*/.63* |
| Optimism | -.06/-.05 | -.05/-.00 | - | .66*/.45* | .72*/.66* | .48*/.53* |
| Self–Esteem | .061/.02 | -.07/-.00 | .11/-.13 | - | .72*/.45* | .60*/.40* |
| Hope | -.13/-.13 | .09/.11 | -.07/-.01 | -.08/-.07 | - | .61*/.64* |
| PANAS_Pos | -.09/-.11 | -.05/-.01 | -.05/-.08 | -.05/-.00 | .03/.01 | - |
| *Note: * p < .001; The correlations for mothers with older children are presented above the diagonal. The correlations for mothers with younger children are presented below the diagonal.* | | | | | | |

**1 Supplementary Figure**


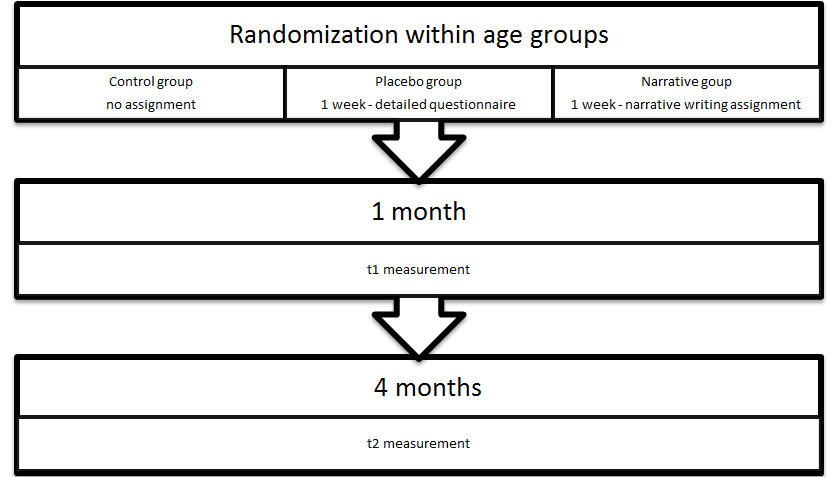


**Supplementary Figure 1 | Detailed experimental design.**
